# Supplementary material for: Resolving the full spectrum of human genome variation using Linked-Reads
Source: Genome Res. 2019 Apr;29(4):635–45. doi: 10.1101/gr.234443.118 (PMC6442396; doi:10.1101/gr.234443.118)
Supplement: Supplemental Material [file supp_gr.234443.118_Supplemental_longranger-2.2.zip › longranger-2.2.tar.bz2!/lr2.2-public/lib/stan/cmdstan/stan/doc/api/html/classstan_1_1io_1_1cmd__line-members.html]

Stan: Member List


|  |  |
| --- | --- |
|  | Stan  2.5.0  probability, sampling & optimization |


- Main Page
- Related Pages
- Namespaces
- Classes
- Files

- Class List
- Class Index
- Class Hierarchy
- Class Members

All Classes Namespaces Files Functions Variables Typedefs Enumerations Enumerator Friends Macros Pages

- stan
- io
- cmd\_line

stan::io::cmd\_line Member List

This is the complete list of members for stan::io::cmd\_line, including all inherited members.

|  |  |  |
| --- | --- | --- |
| bare(size\_t n, T &x) const | stan::io::cmd\_line | inline |
| bare\_size() const | stan::io::cmd\_line | inline |
| cmd\_line(int argc, const char \*argv[]) | stan::io::cmd\_line | inline |
| command() | stan::io::cmd\_line | inline |
| has\_flag(const std::string &flag) const | stan::io::cmd\_line | inline |
| has\_key(const std::string &key) const | stan::io::cmd\_line | inline |
| print(std::ostream &out) const | stan::io::cmd\_line | inline |
| val(const std::string &key, T &x) const | stan::io::cmd\_line | inline |


---

[ Stan Home Page ]

*© 2011–2014,
Stan Development Team.*
